# Reducing the number of EMG electrodes during online hand gesture classification with changing wrist positions

**DOI:** 10.1186/s12984-022-01056-w

**Published:** 2022-07-21

**Authors:** Luis Pelaez Murciego, Mauricio C. Henrich, Erika G. Spaich, Strahinja Dosen

**Affiliations:** Neurorehabilitation Systems, Department of Health Science and Technology, Aalborg University, Aalborg, Denmark

**Keywords:** Surface electromyography, High-density electrodes, Human–machine interfaces, Gesture recognition, Hand rehabilitation, Rehabilitation robotics

## Abstract

**Background:**

Myoelectric control based on hand gesture classification can be used for effective, contactless human–machine interfacing in general applications (e.g., consumer market) as well as in the clinical context. However, the accuracy of hand gesture classification can be impacted by several factors including changing wrist position. The present study aimed at investigating how channel configuration (number and placement of electrode pads) affects performance in hand gesture recognition across wrist positions, with the overall goal of reducing the number of channels without the loss of performance with respect to the benchmark (all channels).

**Methods:**

Matrix electrodes (256 channels) were used to record high-density EMG from the forearm of 13 healthy subjects performing a set of 8 gestures in 3 wrist positions and 2 force levels (low and moderate). A reduced set of channels was chosen by applying sequential forward selection (SFS) and simple circumferential placement (CIRC) and used for gesture classification with linear discriminant analysis. The classification success rate and task completion rate were the main outcome measures for offline analysis across the different number of channels and online control using 8 selected channels, respectively.

**Results:**

The offline analysis demonstrated that good accuracy (> 90%) can be achieved with only a few channels. However, using data from all wrist positions required more channels to reach the same performance. Despite the targeted placement (SFS) performing similarly to CIRC in the offline analysis, the task completion rate [median (lower–upper quartile)] in the online control was significantly higher for SFS [71.4% (64.8–76.2%)] compared to CIRC [57.1% (51.8–64.8%), p < 0.01], especially for low contraction levels [76.2% (66.7–84.5%) for SFS vs. 57.1% (47.6–60.7%) for CIRC, p < 0.01]. For the reduced number of electrodes, the performance with SFS was comparable to that obtained when using the full matrix, while the selected electrodes were highly subject-specific.

**Conclusions:**

The present study demonstrated that the number of channels required for gesture classification with changing wrist positions could be decreased substantially without loss of performance, if those channels are placed strategically along the forearm and individually for each subject. The results also emphasize the importance of online assessment and motivate the development of configurable matrix electrodes with integrated channel selection.

## Background

Myoelectric control is an attractive method for human–machine interfacing with potential applications in multiple domains. It allows translating electrical muscle activity recorded using electromyography (EMG) into control commands for different devices, from general-purpose consumer electronics to assistive systems for the restoration of function and rehabilitation [1]. In recent years, the design of compact multichannel EMG interfaces that are convenient for daily life applications (e.g., implementing dry electrodes) has gained significant attention from both industry and academia [2], bringing successful commercial solutions to the market. Some examples are the Myo-armband (Thalmic Labs, USA) and its more advanced successor from CTRL-Labs (Facebook, USA) for general-purpose applications or the more specialized COAPT (COAPT LLC, USA) system, designed for upper-limb prosthesis control. Furthermore, recent studies demonstrated that post-stroke paretic patients might benefit from the use of pattern recognition of surface electromyographic (EMG) signals to control assistive devices for clinical neuro-rehabilitation [3, 4].

The myoelectric control based on pattern recognition relies on the ability of the user to produce distinguishable and repeatable contractions [2]. Surface EMG recorded during those contractions is used to train machine-learning algorithms to recognize the patterns and generate motion predictions based on muscle activity acquired online. Despite the high classification accuracy presented in the literature (> 90%), pattern recognition-based solutions still lack robustness in daily life applications [5]. The classification performance is sensitive to multiple factors such as contraction levels [6], muscle fatigue [7], arm positions [8], and electrode shift [9], which affect the underlying muscle activity and change the patterns during online use with respect to those recorded during training.

One such factor that is known to affect the performance of a hand gesture classifier is the orientation of the forearm [10–12]. Since the muscles responsible for wrist and finger motion reside within the same forearm compartments, any EMG activity generated during the wrist movement may interfere with the signals produced during hand gestures. Furthermore, the changes in forearm orientation can affect the relative position of the muscles with respect to surface electrodes. Moving the wrist while performing gestures is a common activity in daily living and hence, a robust myocontrol strategy should allow wrist movements without compromising gesture classification accuracy. In [12], the authors found that wrist rotations produced a displacement of the “center of gravity” of the forearm muscle maps. In [10], the authors evaluated the effect of wrist position on pattern classification and found that including data from different wrist positions improves the generalization of the classifier. A recent study demonstrated that training the classifier using kinematic information from the wrist joint together with EMG recorded from intrinsic hand muscles, could reduce the classification error of several hand motions [11]. However, none of these studies evaluated the influence of the electrode set on the classification accuracy when multiple wrist positions are considered in the training data.

Electrode location plays an important role in achieving a robust myocontrol performance [13, 14]. A well-established approach to recording EMG for upper limb myoelectric control is to distribute a few electrodes equidistantly around the proximal forearm. The uniform placement provides an acceptable trade-off between ease of use (simple configuration) and performance [13, 15, 16]. However, such placement might not properly capture muscle activity patterns due to varying wrist positions compared to more targeted positioning. Moreover, the skin areas from where strong muscle activity (“hot spots”) is recorded while performing different gestures may change between individuals due to anatomical differences. Therefore, the same electrode distribution might not be optimal for all subjects. Alternatively, a common approach to improve myocontrol performance is to place the electrodes manually by a clinical expert based on palpation, established guidelines (e.g., SENIAM), and/or biomechanical knowledge [17]. Finally, recording from a larger surface of the skin using High-Density EMG (HD-EMG) allows for capturing high-fidelity spatial information regarding muscle activity without expert intervention [18]. This last approach could be used to determine individually for each subject the electrode locations where the strongest muscle activity (topographical “hot spots”) is observed during movements.

Although the use of HD-EMG in motion classification provides high performance [19–21], increasing the number of channels adds complexity to the overall system. Reducing the number of channels decreases the number of input features for classification, thereby simplifying the processing and decreasing the likelihood of overfitting. Combined with “configurable” electrodes that allow channel selection, this could also decrease the complexity of the amplifier used to record the data [22, 23]. The electrode channels relevant for classification can be selected automatically using feature selection methods [14, 24–26]. In [24], the authors presented an offline performance comparison between methods for electrode reduction using common spatial filtering and sequential forward selection (SFS). The classification accuracy achieved with the two methods was not significantly different. A more recent study demonstrated similar classification accuracy in gesture recognition between channels selected by SFS compared to a selection method based on Fisher’s class separability index [27].

As explained above, wrist orientation might affect the spatial distribution of muscle activity generated while performing gestures. In turn, this can change the electrodes that are relevant for hand gesture recognition. However, none of the aforementioned studies investigated the combined impact of channel configuration and wrist position. The present study, therefore, aims at investigating whether the classification performance can benefit from the use of targeted electrode placement that is robust to different wrist orientations, during online control. We explored the impact of varying wrist orientations on the configuration of selected electrodes in terms of their location, number, and consistency across subjects. Previous research has shown that offline analysis is not a good predictor of online performance [28], and hence the present experiment included an online control task. We hypothesized that a set of strategically placed electrodes would outperform the same number of electrodes placed uniformly along the circumference of the forearm.

## Methods

### Participants

Thirteen right-handed able-bodied subjects (29 ± 3.1 years) participated in the study. Oral and written information was provided to the participants before starting the experimental session and they signed the informed consent form. The study was conducted following the Declaration of Helsinki. According to the Danish Act on Research Ethics Review of Health Research Projects, the study did not require approval from the Research Ethical Committee (Journal number: 2019-000199).

### Experimental setup

**Fig. 1:**
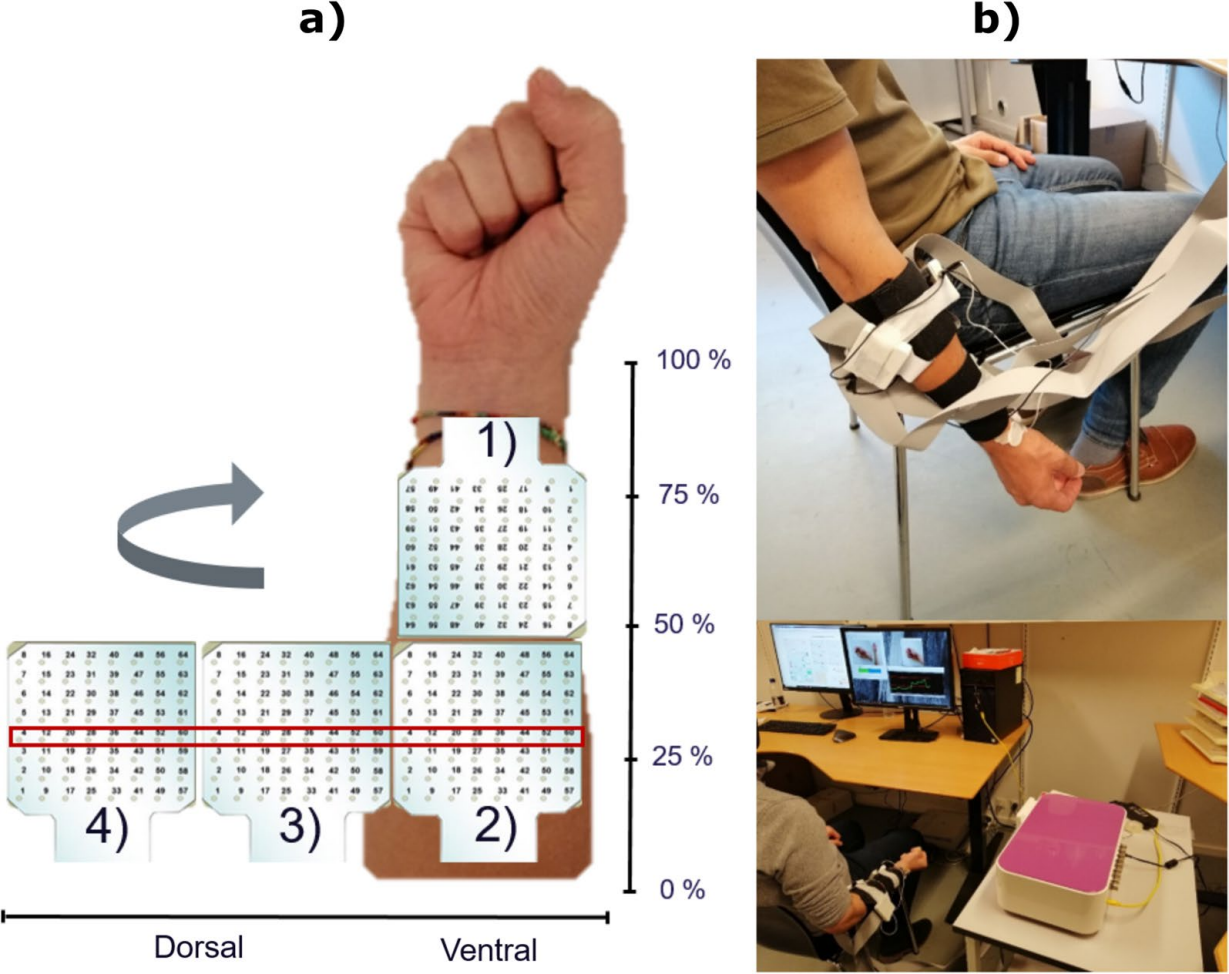
Experimental setup. **a** Placement of the high-density electrode grids on the forearm of a participant. One electrode grid was placed on the ventral side of the distal forearm (grid 1) and three grids were positioned around the proximal forearm (grids 2 to 4). A red rectangle indicates the row of channels selected for uniform placement. **b** Lateral view of a participant with the arm in resting position (top) and with the elbow flexed at 90° (bottom) during the experimental session

Participants sat comfortably on a chair in front of the computer screen, with the elbow flexed at approximately 90° (Fig. 1b, bottom panel). The skin of the forearm was previously cleaned and shaved. Four electrode grids (GR10MM0808, OTBioelettronica, 8 × 8 channels, 10 mm inter-electrode distance) were used to record EMG signals from 256 channels in a monopolar configuration from the right forearm (Fig. 1a). Three of them were mounted in a circumference around the forearm, at approximately 20% of the forearm length distally from the elbow crease. The fourth electrode grid was placed consecutively on the anterior forearm. This electrode configuration allowed acquiring EMG activity from the muscles responsible for finger and wrist motion. Reference and ground self-adhesive Ag/AgCl electrodes (Neuroline 720, Ambu, Denmark) were placed on the wrist, over the radial styloid process, and on the elbow, over the olecranon process of the ulnar bone, respectively. The EMG was recorded using a multichannel bio-signal amplifier (Quattrocento, OTBioelettronica, Italy) connected to the host computer via Ethernet. The signals were band-pass filtered through the built-in filters (2nd order Butterworth, bandwidth 10–500 Hz) and sampled at 2048 Hz.

### Experimental protocol

**Fig. 2:**
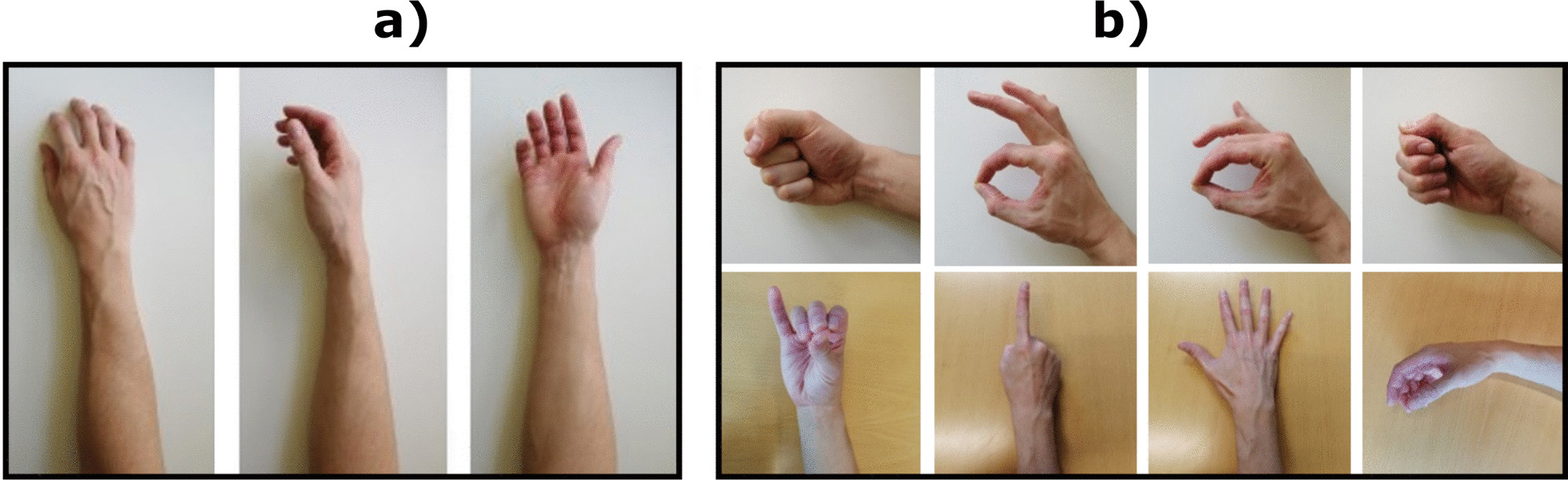
Experimental task. The participants performed eight gestures in three wrist positions: **a** The three wrist positions (full pronation, neutral, full supination). **b** Eight gestures (from left to right and top to bottom: power grasp, one-finger pinch, two-fingers pinch, lateral grasp, three-fingers pinch, index pointing, hand open and rest). Each gesture was performed in all wrist positions at two muscle contraction levels

The experimental task (Fig. 2) was to classify 8 hand gestures (power grasp, one-finger pinch, two-fingers pinch, three-fingers pinch, lateral grasp, index pointing, hand open, and rest) in 3 wrist orientations (full supination, neutral, full pronation) and two muscle contraction levels (low and moderate). Training the classifiers with data collected at different muscle contraction levels is a common approach to provide proportional control during pattern recognition-based myocontrol [29, 30].

**Fig. 3:**
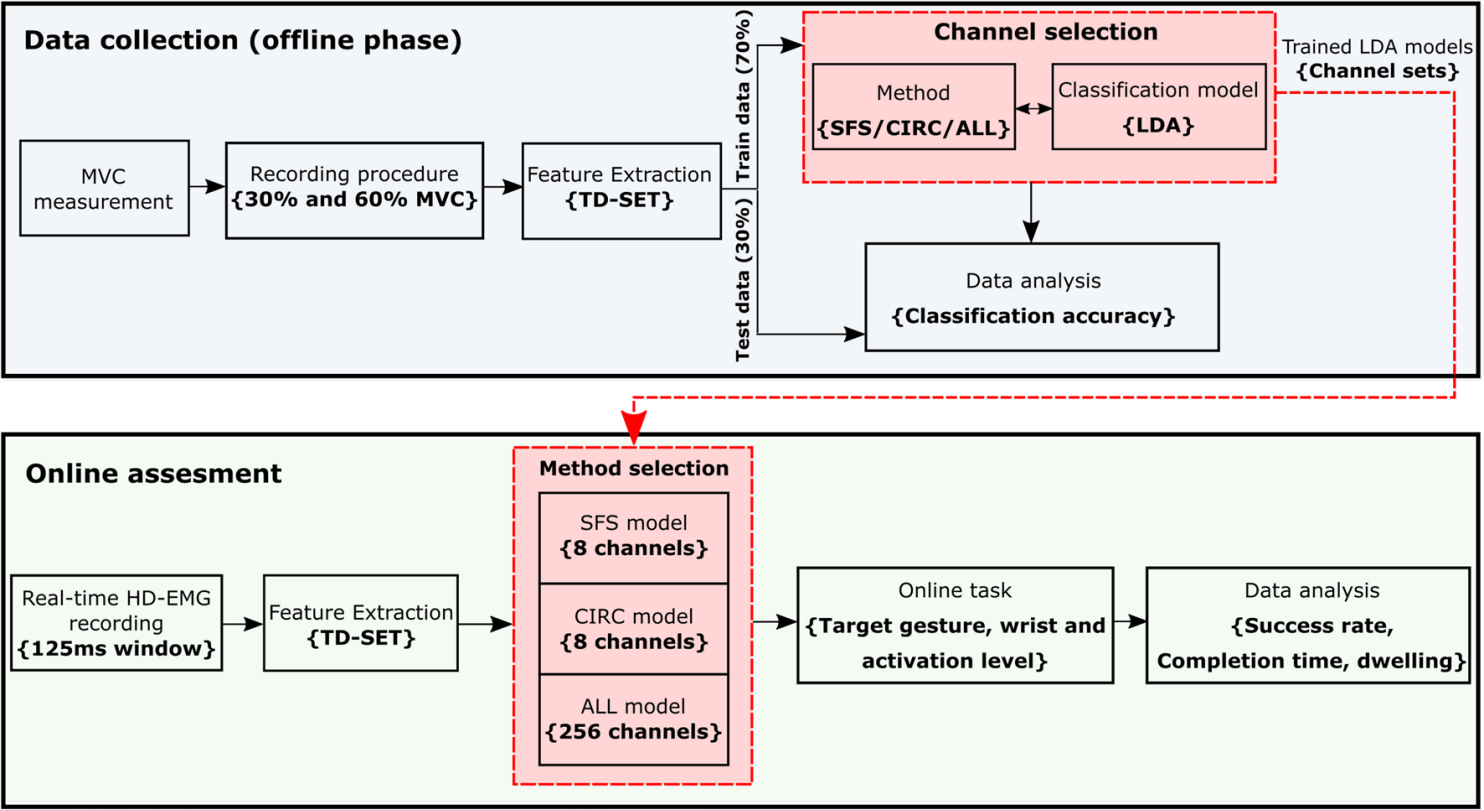
Flow diagram of the experimental protocol. The experiment was divided into two phases: data collection (offline phase) and online assessment. During the offline phase, HD-EMG data was collected and features were extracted. After training the classifiers for different number of channels selected using SFS, CIRC and ALL, the offline classification accuracy was calculated using testing data. Once the offline analysis was completed, three electrode configurations (SFS, CIRC and ALL) were evaluated during an online gesture recognition task

The flow chart of the experimental protocol is shown in Fig. 3. The experimental protocol included two phases: data collection and online assesment. Offline analysis was conducted to select a subset of electrodes (see Channel selection) and assess the classification performance for different numbers of electrodes. In the online test, two electrode configurations with 8 channels as well as a full set of electrodes were compared during online control. A graphical user interface (GUI) was developed to guide the participants through the experiment.

Visual inspection of the EMG signals was conducted at the beginning of the experiment to remove channels corrupted by noise due to poor electrode-skin contact (e.g., usually up to 4 channels per subject). The identified channels were labelled as “corrupted” and discarded from further analysis.

#### Data collection

Maximum voluntary contraction (MVC) was recorded for each gesture in all wrist positions. Participants were asked to perform a sustained muscle contraction of the gesture prompted on the screen at their strongest, but still comfortable force level, and hold it for 3 s. Resting periods of 10 s were inserted between the gestures. The MVC values were calculated as the root mean square (RMS) computed over 250-ms windows of the EMG averaged over the contraction period (3 s) and across all the channels.

##### Recording procedure

**Fig. 4:**
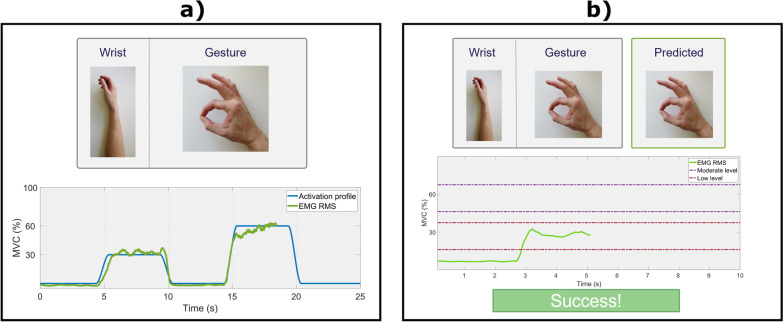
User interface. **a** Task for data collection. Participants were asked to perform the gestures shown by the graphical user interface and follow the muscle activation profile (blue line) by producing two sustained contractions with plateaus at 30% and 60% of the MVC for the respective gesture. The contraction level was estimated as the root mean square of windowed EMG across all the channels and provided as feedback to the user (green line). **b** Online test interface. Participants were asked to match a target gesture and wrist position while the predicted gesture was shown on the side. The task was considered successful if the subject matched the right gesture for two consecutive seconds while maintaining the contraction level inside the range indicated by the corresponding pair of dashed lines (i.e., low or moderate)

HD-EMG recorded during two sustained contractions at low (30% of MVC) and moderate (60% of MVC) levels was acquired for each combination of hand gesture and wrist position. A pseudorandom sequence of 24 tasks, corresponding to each combination of gesture and wrist position (8 gestures × 3 wrist positions), was shown to the participant through the GUI (Fig. 4a). Once the task was prompted, the subject had 5 s to rotate the wrist comfortably to the position indicated on the screen before the recording started. The participants used the overall level of muscle activation to control the amplitude of the signal (green line in Fig. 4a) along the vertical axis, while the signal moved automatically along the horizontal axis. The muscle activation level was estimated by computing the RMS of the EMG in 250-ms windows for each channel and then averaging across channels. The participants were asked to modulate their muscle contraction so that the generated signal tracked the reference muscle activation profile (blue line in Fig. 4a). The activation profile comprised two trapezoids with 1-s ascending-descending slope, 4-s plateau, and 5-s separation. The plateaus were set at 30% and 60% of the MVC for the first and second trapezoid, respectively. The tasks were executed sequentially with 15-s rest between the tasks.

##### Feature extraction

After the data were collected, the EMG was segmented to include only those portions that corresponded to contractions. The contraction onset was defined by detecting when the muscle activity crossed the threshold of 10% MVC. For the rest class, 4 s of data recorded during hand resting periods were used for the analysis. The extracted segments (8 hand gestures × 2 force levels × 3 wrist positions) were concatenated and labeled accordingly. Five time-domain (TD) features: mean absolute value (MAV), zero crossing (ZC), slope sign change (SSC), wavelength (WL), and the logarithm of variance (LogVar), were extracted in sliding windows of 250 ms with 50% overlap. These features have been commonly used in literature for recognizing hand motions [1, 31]. The resulting dataset was randomly divided into a training (70%) and a testing dataset (30%) using a stratified holdout method.

##### Channel selection

The channel selection with SFS was performed using the training dataset. The SFS method started with an empty set of features and sequentially added new features to increase the classification accuracy. In the present study, the classification was implemented using linear discriminant analysis (LDA), which has effectively become the golden standard in myocontrol for both academic and commercial applications. The performance of each candidate feature was validated through tenfold cross-validation. To obtain the channel subset, each of the features selected by the SFS was mapped back to the parent channel. As more than one feature may belong to the same channel, the final number of selected channels could be smaller than the number of selected features. To test the performance for the given number of selected channels (see Data Analysis), new features were sequentially added until the desired number of channels was reached for each subject. Once the desired number of channels was selected, all five features extracted for those channels were used in the further analysis. The focus of the present study was on selecting the channels (electrode pads) rather than individual features. Therefore, we followed a common practice in myoelectric control and used a well-established set of time-domain features [32] associated with each selected channel. This approach might not be optimal since some of the extra features might have a lower separability score. However, fixing the number of features across the methods allowed us to highlight the relevance of the channel locations for gesture classification.

Three methods to select the location of the electrodes from the HD-EMG interface were compared: the aforementioned sequential forward selection (SFS), the circumferential evenly distributed placement (CIRC), and the full set of electrodes (ALL). The SFS method provides a subject-specific channel set based on the discriminative power of their features, while the CIRC method is a standardized uniform distribution “blind” to the muscle characteristics. Both methods were compared to ALL as a benchmark. For CIRC, the channels from the fourth row of the proximal matrices arranged equidistantly and circumferentially around the subject's forearm were chosen (Fig. 1a). If any of the channels included in the CIRC set was previously labelled as “corrupted”, the channel located immediately below was selected instead.

The resulting set of features obtained from the selected channels was then used to train an LDA classifier for recognizing 8 gesture classes. The classifier was trained using the features extracted from the training dataset and its performance was assessed by applying the trained classifier to the testing data. Depending on the analysis performed (see Data analysis), the features extracted from the EMG corresponding to the same gesture at two contractions levels and in different wrist positions were labelled as the same gesture class. For example, when all wrist positions were considered for the analysis, the training samples labelled as gesture 1 (power grasp) contained the features from the EMG recorded during power grasp contractions at two force levels and three wrist positions. Similarly, when the analysis was conducted over a single wrist position (e.g., pronation), the training samples for gesture 1 comprised the features from power grasp at two contraction levels with the wrist pronated.

#### Online assessment

Based on the results of the offline analysis (pilot tests), three channel configurations were selected to be tested in the online experiment: 8 channels selected using CIRC, 8 channels selected using SFS, and the full set of channels (benchmark). The classification models for online control were trained using all collected data.

The online classification pipeline is illustrated in Fig. 3 (bottom box). HD-EMG was recorded in windows of 125 ms and concatenated with 50% of the data from the previous window. TD features were then extracted from the resulting 250 ms segment of EMG. A selected number of features was used as the input of the classifier based on the channel selection method that was active in the specific trial of the grasp recognition task. When the method ALL was active, all features (256 channels × 5 TD features) were fed into the classifier. In case CIRC or SFS was selected, only the features corresponding to the respective channel sets (8 channels × 5 TD features) were used for classification. Participants were unaware of which method was active during the online trials.

A GUI was developed to guide the participants during the online control task (Fig. 4b). At the beginning of each trial, the subject was shown the position of the wrist in the upcoming task for 5 s. During that time, they positioned the wrist accordingly. After the brief rest time (5 s), the target gesture was displayed. The target task (i.e., a combination of gesture and wrist position) and the online prediction of the trained classifier (a new prediction every 125 ms) were shown in the top part, while the bottom part displayed the estimated muscle activation level and the target activation window. The subject was asked to perform the indicated movement so that the prediction of the classifier matched the target gesture continuously during 2 s (dwell time). The online prediction was shown only while the muscle activation level was maintained within the target window; otherwise, a notification message indicated to the subject to correct the activation level. The online feedback allowed the subject to notice a wrong classification and potentially change how they performed the movement. The subject had 10 s to complete the task; otherwise, the task was considered failed. The success and failure were indicated with auditory and visual cues.

The online experiment was divided into two blocks. Each block included one repetition of each combination of hand gesture, wrist position, and channel set (CIRC, SFS, and ALL) in random order (i.e., 8 gestures × 3 wrist positions × 3 methods). The participants were asked to repeat each block at low (target window 10–35% MVC) and moderate force (target window 45–75% MVC). The order of the blocks was randomized.

### Data analysis

The effect of wrist position on the complexity of the classification task was evaluated by computing the Bhattacharyya distance as the index of separability between the classes [33]. The Bhattacharyya index was calculated as the mean distance across all pairs of classes.

The outcome measure in the offline analysis was the classification accuracy of hand gestures using data from both contraction levels combined. The classification accuracy was computed when the classifier was trained and tested using the data from (1) each single wrist position and (2) all wrist positions combined. The classification accuracy reported for single wrist conditions was calculated as the average accuracy across the three wrist positions. Additionally, a comparison of classification accuracies between the electrode selection methods (SFS, CIRC, and ALL) was conducted separately for single and combined wrist positions across the different numbers of electrodes.

The outcome measures to evaluate the online control were completion rate, completion time, and the number of dwellings. The completion rate was calculated as the number of successfully completed trials over the total number of trials. The completion time and the number of dwellings were computed from the successful trials only. Completion time was defined as the time needed to complete the task. The number of dwellings quantified the stability of control and was defined as the number of times that the classifier output changed the prediction while the participant maintained the contraction level within the target window.

The normality of the data distribution was assessed using the Shapiro–Wilk test. When data sets were non-normally distributed, the non-parametric Friedman’s test was used. Post hoc pairwise comparisons were performed using the Wilcoxon Signed-Rank test and corrected for multiple comparisons using Bonferroni correction. For normally distributed data, repeated measures analysis of variance (RM-ANOVA) was used. Paired t-tests were conducted for pairwise comparison and adjusted for multiple comparisons with Bonferroni correction. The significance threshold was set at p < 0.05.

## Results

### Impact of wrist position on classification complexity

**Fig. 5:**
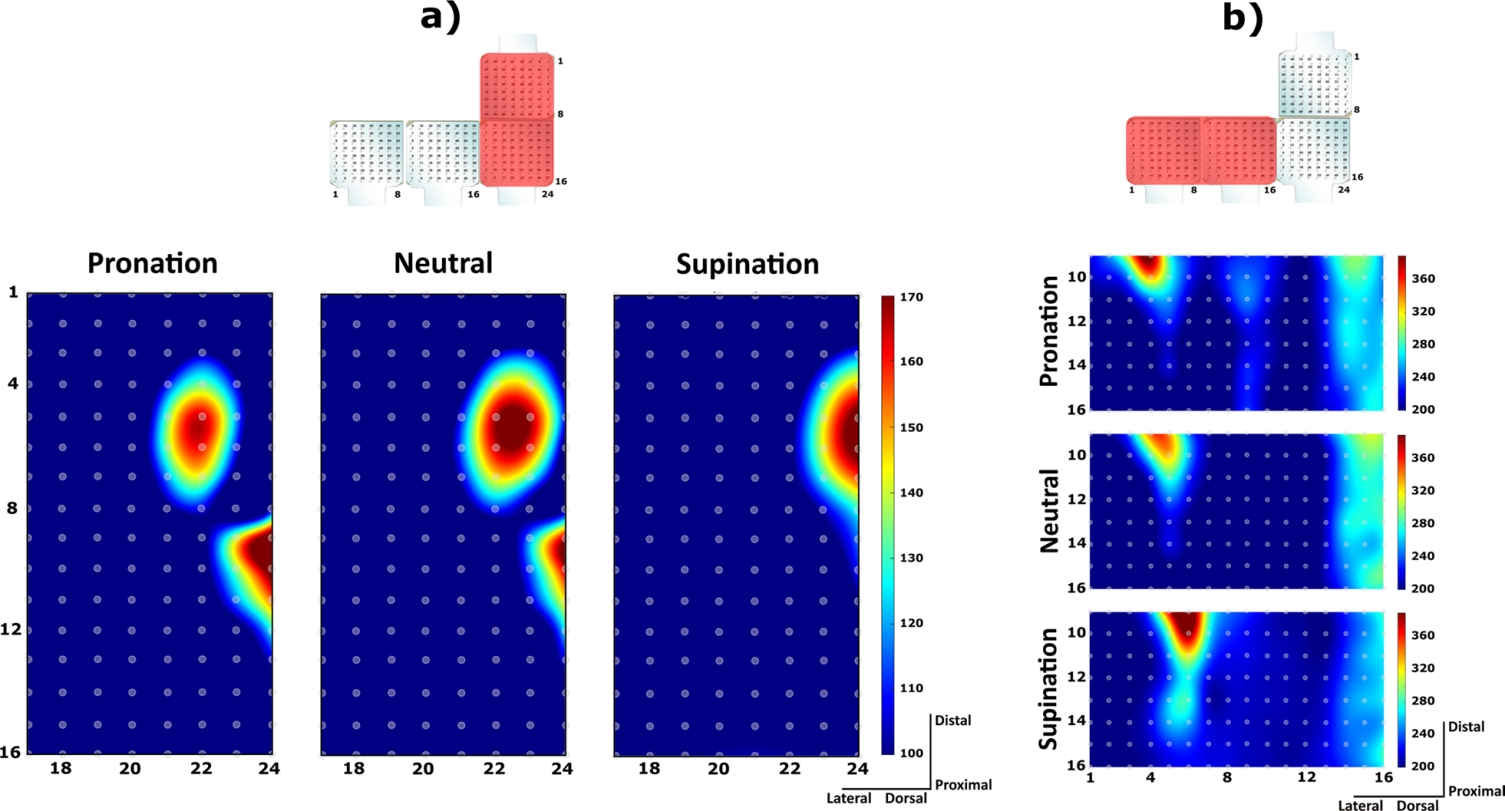
Representative muscle maps obtained by interpolation of muscle activity recorded using HD-EMG. Each grey circle indicates an electrode from the grid and the color shows the root mean square (RMS) of the EMG in μV, over a 250-ms data window (see color legend). **a** Maps generated from to the two matrices placed distally and proximally over the ventral side of the forearm (flexors). Data were recorded from a subject performing a sustained lateral grasp in the three wrist positions. **b** Maps corresponding to the two matrices placed over the dorsal and lateral side of the proximal forearm (extensors). Data recorded from the subject performing a sustained index pointing in the three wrist positions. Varying the position of the wrist produced different spatial distributions of muscle activity for the same hand gesture

Figure 5 illustrates the effect of wrist position on the spatial pattern of muscle activity captured using HD-EMG. In the example, a representative subject performed two gestures: lateral grasp (Fig. 5a) and index pointing (Fig. 5b), in all wrist positions. For both hand gestures, the localized areas of muscle activity shifted visibly with respect to the surface electrodes when the position of the wrist changed from full pronation to full supination. During lateral grasping, the muscle “hot spots” recorded by the volar matrices gradually moved towards the medial aspect of the forearm. During pointing, the activity was concentrated in the dorsal and lateral matrix, and the “hot spots” shifted more proximally.

**Fig. 6:**
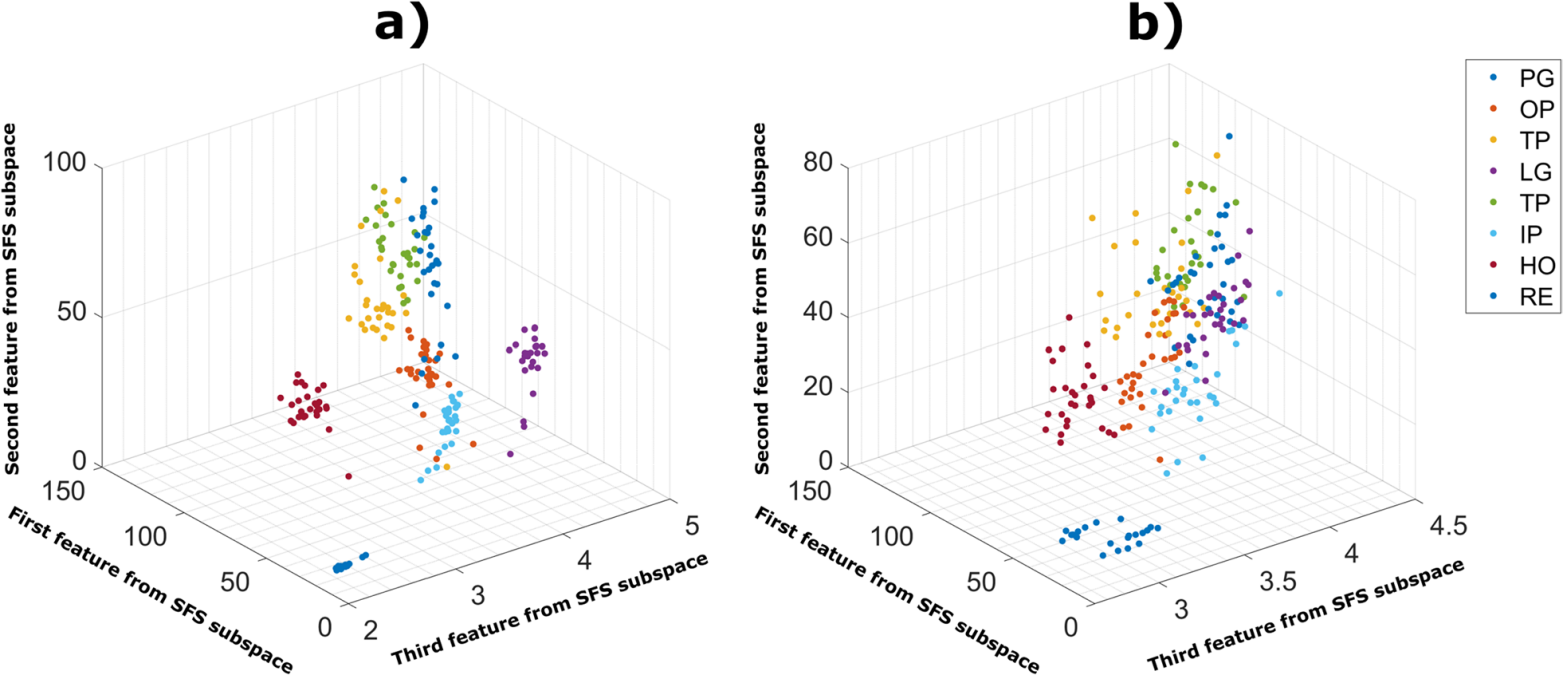
An illustrative example of the effect of wrist position on the separability of 8 classes in the projected feature space. **a** Scatter plot of the first three principal components of the feature vectors when including data in neutral position. **b** Scatter plot of the first three principal components when considering data from all wrist positions. *PG* power grasp, *OP* one-finger pinch, *TP* two-fingers pinch, *LG* lateral grasp, *TP* three-fingers pinch, *IP* index pointing, *HO* hand open, *RE* hand rest

The previous examples show that the changes in the muscle maps produced by the wrist rotations can increase the variability inside the gesture class. Indeed, when the data from all wrist positions were considered in the analysis, the separability between the clusters of features decreased as illustrated in Fig. 6. The SFS features projected to the 3D space using principal component analysis exhibited significant overlap when data from all wrist positions were included (Fig. 6b). The feature clusters are however well separated when using only the data from the wrist in the neutral position (Fig. 6a).

**Fig. 7:**
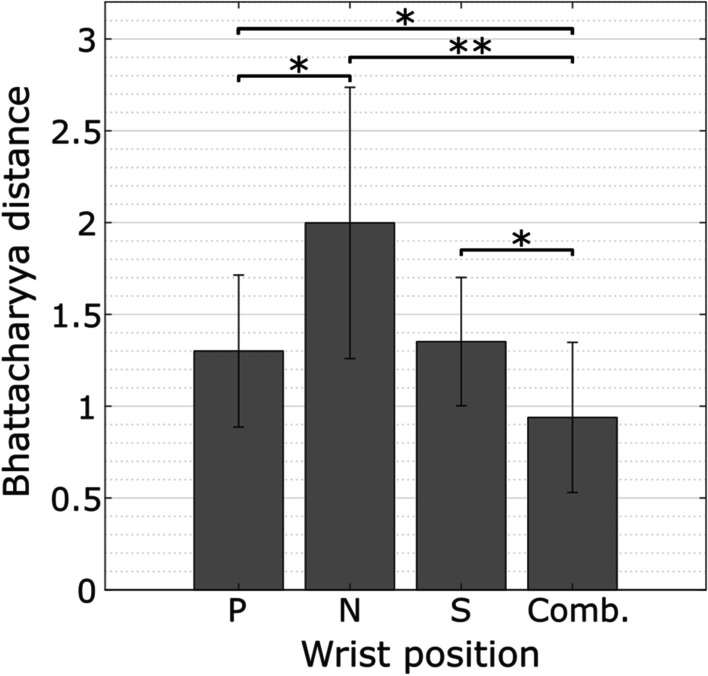
Separability index between gesture classes computed using Bhattacharyya distance for multiple classes. The distances were calculated from the SFS feature space using data from different wrist positions (single: P (pronation), N (neutral), S (supination); and all combined: comb)

To quantify the impact of the wrist position on the complexity of classification, the Bhattacharyya distance between classes in the SFS feature space was calculated for all wrist positions (Fig. 7). Statistical analysis showed a main effect (χ^2^ = 18.6, p < 0.001) of the wrist position. The distance between classes was significantly lower (p < 0.05) when using data combined from all wrist positions compared to using data from any single wrist position. In particular, the difference was largest (p < 0.01) when comparing data from the neutral position to all combined. Between single wrist positions, the distance was significantly larger (p < 0.05) when using data from the neutral position compared to pronation. No significant difference in the distance was found between supination and pronation.

### Offline performance

**Fig. 8:**
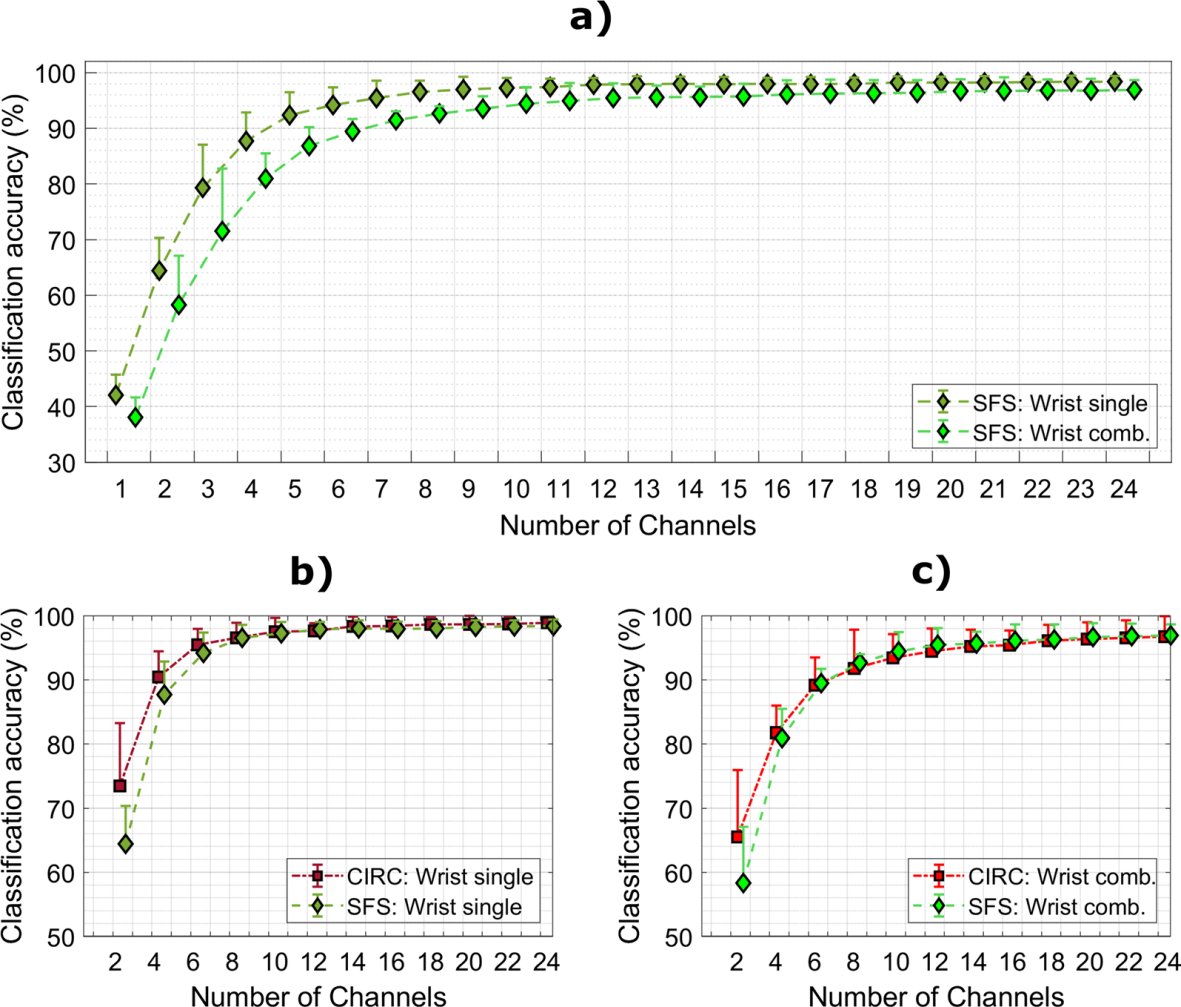
Classification performance for different number of channels. **a** Classification accuracy for the channels selected using SFS. **b** Comparison between electrode selection methods using data from single wrist positions and **c** using data from three wrist positions combined. “Wrist single” corresponds to the average classification accuracy obtained when training and testing the classifier using data from single wrist positions. “Wrist comb.” is the average classification accuracy when the classifier was trained and tested using data from the three wrist positions combined

Figure 8 shows the average classification accuracy for the different numbers of channels for single and combined wrist positions (Fig. 8a) when using CIRC and SFS channel selection methods (Fig. 8b and c). Expectedly, as the number of channels (n) increases, the accuracy improves monotonically until reaching a plateau (n > 8). The maximum accuracy in all conditions (> 98%) was achieved when using the full electrode set (ALL). Nevertheless, high accuracy (> 90%) could be achieved in all cases with a substantially smaller number of electrodes (n > 7). The statistical analysis revealed that including the data from the 3 wrist positions combined, significantly decreased the accuracy of both channel selection methods. More specifically, when using SFS there was a significant difference (p < 0.05) in performance between single versus combined conditions when more than 4 channels were used (except for 17, 18, and 21 channels). Similar results were obtained with CIRC where the accuracy was significantly higher (p < 0.05) for single wrist condition regardless of the number of channels.

Additionally, when comparing the classification accuracy of SFS versus CIRC, no statistically significant difference was found for any number of channels for single (Fig. 8b) and combined (Fig. 8c) wrist positions. Moreover, the classification accuracy was similar when the classifier was trained and tested using data from combined wrist conditions at a single contraction level (low and medium), with no statistical difference between the two methods.

**Fig. 9:**
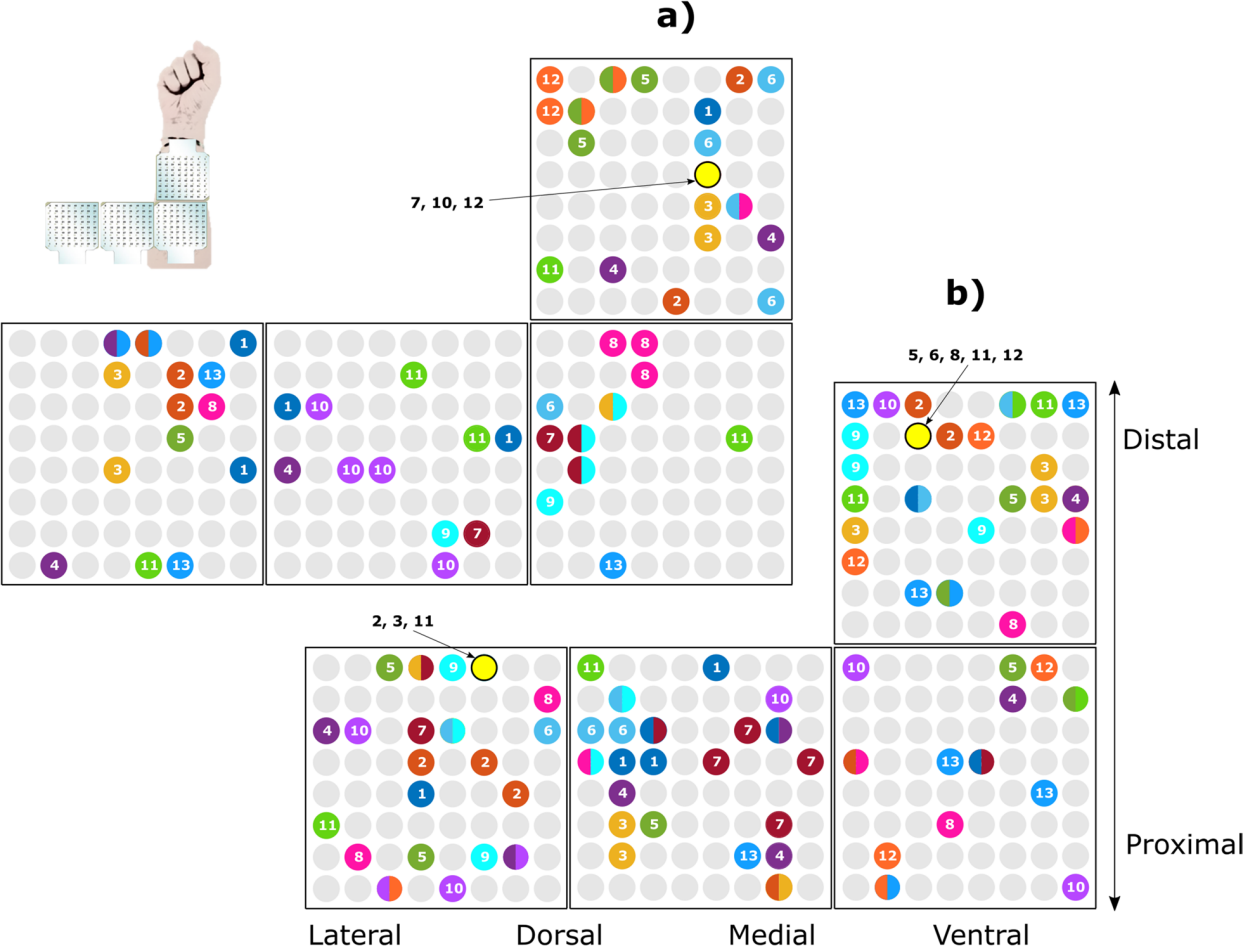
The channels selected using SFS across all participants. The distribution of the matrices over the forearm is shown in the icon on the top-left corner. The top square represents the distal matrix, and the 3 bottom squares represent the three matrices placed around the proximal forearm. Colored circles are the selected channels. Each participant is represented by a different color and the participant number is indicated inside the circle. For channels selected for 2 participants, the circle is filled by the colors assigned to the respective participants. In case where more than 2 participants share the same channel, the participant numbers are indicated with an arrow (yellow circles). **a** Five channels selected across participants when the classifier was trained in the neutral wrist position. **b** Eight channels selected across participants when the classifier was trained using the data from all wrist positions combined

Figure 9 illustrates the channels selected using SFS across all participants when the classifiers were trained using the data from a single position (neutral, Fig. 9a) and all wrist positions combined (Fig. 9b). The two-channel sets resulted in similarly high performance (mean ± SD) in the two conditions, i.e., 92.3 ± 6.5% for 5 channels versus 92.4 ± 1.5% for 8 channels. Importantly, the distribution of the channels was highly subject-specific, as most of the channels (46 channels for wrist single and 60 channels for wrist combined) were unique to a single participant, while only a few of them (9 channels and 18 channels) were selected in two or more participants. Therefore, the channels with discriminative information differ substantially between participants. The distribution of the selected channels was not uniform across the recording surface of the forearm. When all wrist positions were included in the training data, channels from the two electrode grids placed from the lateral to the medial side of the forearm were selected similarly (50 times) compared to the two matrices placed over the ventral side of the forearm (54 times). In addition, the channels located on the distal electrode placed on the ventral side were selected more often (33 times) than the channels from the electrode grid placed on the same side but proximally (17 times).

### Online performance

**Fig. 10:**
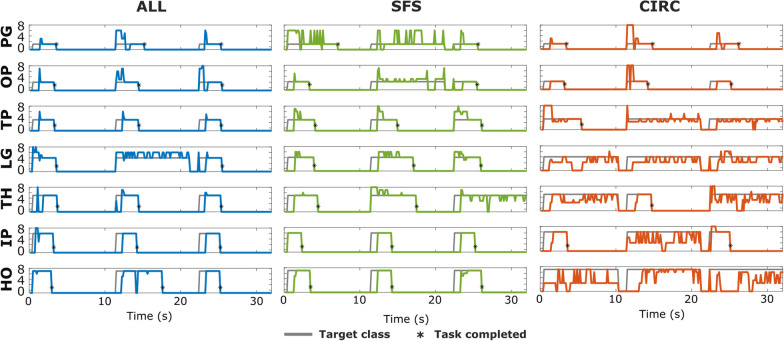
Online classification results at the low contraction level from a representative subject. The colored lines represent the classification output (each 125 ms), while the grey line indicates the target class. Each row corresponds to a different gesture, and includes three consecutives tasks, one in each wrist position (randomized order). Black asterisks indicate the tasks that were completed successfully. *PG* power grasp, *OP* one-finger pinch, *TP* two-fingers pinch, *LG* lateral grasp, *TH* three-fingers pinch, *IP* index pointing, *HO* hand open

Figure 10 illustrates the quality of online predictions from one representative participant using the three channel selection methods (ALL, SFS, and CIRC). When the subject used the full set of channels (ALL), most tasks (20 out of 21) were successfully completed (Fig. 10, left) and the prediction profiles (blue line) matched very well the target class profiles (gray line). A small delay at the beginning of each task corresponds to the subject’s reaction time. After the delay, the participant successfully produced and maintained the target class with a few misclassified samples. With SFS, the participant completed a similar number of tasks (18 out of 21) with similar prediction profiles, though it seems that the “power grasp” was somewhat more challenging for the subject.

In the case of the CIRC method, the classification accuracy decreased substantially as indicated by the frequent oscillations in the prediction profiles. Hence, the subject had difficulties generating as well as maintaining the target class. Overall, he/she completed only 10 out of 21 tasks and without any successful trials for two of the target gestures (i.e., lateral grasp and hand opening). The inability to successfully complete any trial on at least two or more gestures was commonly observed in other participants when using CIRC (9 out of 13 participants), while this was much less frequent when using the other two methods (1 out of 13 participants using SFS and 2 out of 13 participants using ALL).

**Fig. 11:**
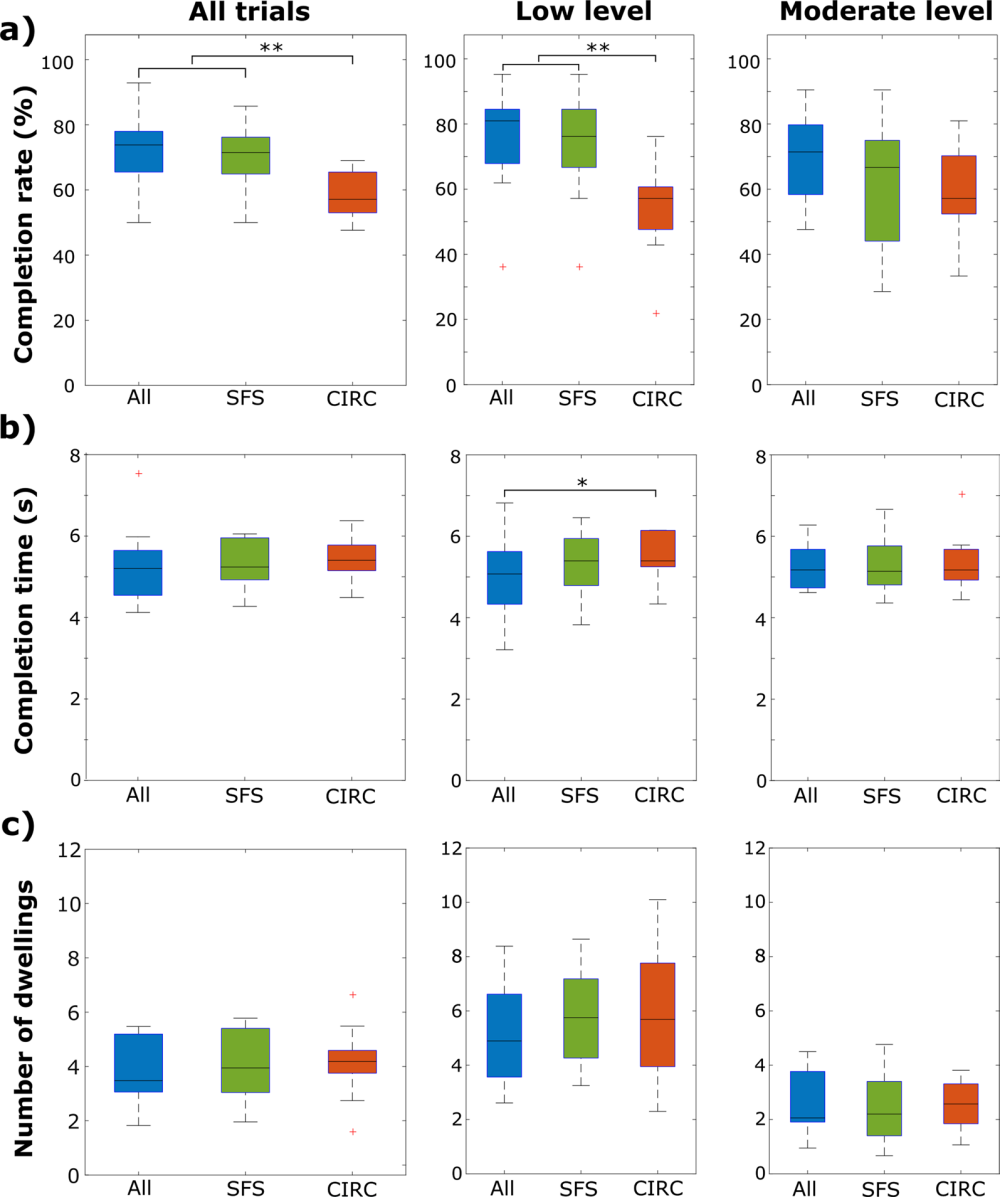
Summary results of the online experiment. The boxplots show the distribution of **a** completion rate, **b** completion time and **c** the number of dwellings over all trials, and separately over the trials at low and moderate contraction levels. Outliers are indicated with “+” symbol. (*p < 0.05, **p < 0.01)

Figure 11 shows the summary results of the online tests for the three channel selection methods grouped by contraction levels. A significant main effect of electrode configuration was found [F(2,16) = 12.8; p < 0.001] on the completion rate computed when both contraction levels were considered. Statistical analysis showed that the completion rate [median (lower–upper quartile)] obtained using SFS [71.4% (64.8–76.2%)] was significantly larger (p < 0.01) compared to CIRC [57.1% (51.8–64.8%)]. Moreover, the completion rate achieved using CIRC was significantly smaller (p < 0.01) compared to ALL [73.8% (65.4–77.9%)]. A significant interaction was found between muscle contraction level and the channel selection method [F(2,16) = 4.4; p < 0.03]. Analysis of simple main effects showed a significant effect of the channel selection method only for the low contraction level [F(2,20) = 19.9; p < 0.001]. Post hoc tests revealed that at low contraction level, the accuracy using CIRC [57.14% (47.6–60.7%)] was significantly lower (p < 0.01) compared to ALL [80.95% (67.8–84.5%)] and SFS [76.19% (66.7–84.5%)]. No significant differences were found between SFS and ALL. For the moderate contraction level, the participants achieved a completion rate of 71.4% (58.3–79.8%) using ALL, 66.7% (44.1–75%) using SFS, and 57.4% (52.3–70.2%) using CIRC, with no statistically significant difference in completion rate between the methods. Additionally, no significant difference in completion rate was found when comparing the same method between the two contraction levels.

Overall, the participants took a similar amount of time to complete the tasks using ALL [5.2 s (4.5–5.6 s)], SFS [5.2 s (4.9–5.9 s)], and CIRC [5.4 s (5.2–5.8 s)], without significant difference between the methods when considering the data from both contraction levels. However, a statistically significant difference was found between the electrode configuration methods [χ^2^(2) = 7.8, p < 0.02] when the target contraction level was low. Post hoc analysis showed that for low contraction level, the participants were significantly faster (Z = − 2.5, p < 0.05) using ALL [5.1 s (4.3–5.9 s)] compared to CIRC [5.4 s (5.3–6.1 s)], but no statistical difference was found compared to SFS [5.39 s (4.7–5.9 s)].

Similar to the previous results, the number of dwellings calculated across both contraction levels was similar for ALL, SFS, and CIRC with 3.4 (3.1–5.2), 3.9 (3.0–5.4), and 4.1 (3.8–4.6), respectively. Statistical analysis showed a significant main effect of contraction level [F(1,8) = 52.0, p < 0.001] on the number of dwellings. Overall, the participants had fewer dwellings when the target contraction was at the moderate level, indicating better control stability. The electrode selection method did not have a significant main effect on the number of dwellings for any contraction level.

## Discussion

This study demonstrated that dexterous hand gestures could be successfully decoded during an online control task including different wrist positions and muscle contraction levels using only a small number of EMG channels. The results showed that introducing multiple wrist positions increased the difficulty of the classification task. Consequently, more channels need to be presented as input into the classifier to achieve the same accuracy as when recognizing the same gestures in a single wrist position. Additionally, despite the two channel selection methods (SFS and CIRC) resulting in a similar performance during offline analysis, SFS outperformed CIRC during online control, especially during grasping using low levels of muscle contraction. Finally, the channels selected using SFS exhibited significant inter-subject variability. The insights obtained in the present study are important for the application of myoelectric control in general applications and/or clinical contexts.

### The impact of wrist position in offline analysis

As illustrated in Fig. 5, varying the wrist position changes the spatial distribution of the EMG responses produced by the hand gestures. The muscle activation patterns associated with the same gesture are thus more variable, which increases the spread of the features within individual classes and leads to lower class separability (Fig. 7). In pattern recognition, the separability between classes is highly correlated with classification accuracy [28, 34].

Surprisingly, the classification accuracy in the offline analysis was not significantly affected by the electrode selection method (SFS or CIRC), regardless of the wrist position (single versus combined) and contraction level (low versus medium). Therefore, the offline classification accuracy did not benefit from the more targeted “placement” of EMG channels, as the simple uniform arrangement of the channels around the proximal aspect of the forearm provided similar performance. A direct comparison of the obtained results with those reported in the literature is difficult, because of the differences in the experimental setup, electrode configuration, and/or task (e.g., number of classes, hand gestures, contraction levels, and arm orientation) across studies. Nevertheless, the offline performance in the present study was in line with that reported in the literature, where the mean classification accuracy ranged from approx. 90–96%, using less than 12 electrodes placed around the arm, forearm and hand [10, 11, 24–27, 35, 36]. In contrast to our proposed method, most of these studies included in their analysis the data from the intrinsic muscles of the hand [11, 24–27, 35, 36] and/or wrist kinematic information [10, 11]. Moreover, bipolar disposable electrodes were used in [10, 11, 25, 26, 35, 36] while HD-EMG was employed in only two cases [24, 27]. In [24, 27], the authors achieved high classification accuracy (above 95%) using 10 channels selected from the HD-EMG interface, similarly as in the present study. However, contrary to our work, these studies placed EMG electrodes on both hand and arm muscles, considered only a single force level and did not include an online assessment, nor evaluated proportional control.

Including the data from multiple wrist positions decreased the performance (Fig. 8), as also reported in [25, 36]. Interestingly, in [11], the authors did not find a significant difference in classification accuracy between single and multiple wrist positions. However, the study considered only 4 hand gestures, and hence, the classification task might have been too simple to detect the impact of wrist position. As shown in [26], increasing the number of classes affects the complexity of the classification task and requires more channels to maintain the same performance.

The channels selected using SFS were highly subject-specific, with only a few channels selected for more than one subject (Fig. 9). The selected channels were spread across the matrices, but not uniformly. Interestingly, quite a few channels were selected from the matrices that were placed over the hand and wrist extensor muscles, which means that the classification also used “indirect” information from the muscles that served a supporting role (e.g., extensors stabilized the wrist while flexors controlled finger motion to produce the desired grasp). Further insight regarding the contribution of such supporting/stabilizing muscle activity to the classification performance could be obtained by correlating the observed muscle activity to the muscle biomechanical function using musculoskeletal models [37]; however, this analysis was outside the scope of the present study. In the case of multiple wrist positions, the channels closer to the wrist joint were selected more often compared to those located proximally. This can be explained by the fact that the muscles moving the fingers (i.e., digitorum superficialis and profundus) are located superficially in this area while in the proximal forearm they are located below the muscles flexing the wrist (e.g., flexor carpi radialis).

### Targeted placement improves performance during online classification

Interestingly, despite the electrode selection was not relevant for offline performance, the results of the online test demonstrated that the channels selected using SFS outperformed the same number of channels selected using CIRC. The CIRC configuration has been commonly used in myocontrol applications for hand gesture recognition [38–40]. However, the present study implies that placing the electrodes uniformly around the forearm might not be the best approach when facing a more challenging classification task, such as online gesture recognition with changing wrist positions.

The online experiment emphasized the gap between offline classification accuracy and online control performance, which has been noted in the literature [41]. During online control, the participants received feedback, which they could use to improve their performance within as well as across trials [28]. In the present experiment, the online task might have provided the participants with an opportunity to actively exploit potential intrinsic advantages of the targeted placement to reach overall higher performance. Moreover, when performing gestures during data collection, the participants were guided using a GUI (Fig. 4), and hence a well-defined and controlled procedure ensured that the gestures and thereby the recorded muscle activity was consistent and reproducible. During the online test, however, the participants were free to vary their movements and they were additionally pressured by time. In contrast to the CIRC channels that were confined to a small proximal area of the forearm, the SFS selected the most discriminative channels spread across a wider forearm area. The resulting classifier was, therefore, more robust to the aforementioned variations in the muscle activation patterns during the online assessment.

The difference between SFS and CIRC was particularly pronounced for the low muscle contraction level, where the performance of SFS was similar to that of ALL electrodes and significantly higher compared to CIRC. In general, as the contraction intensity increases, the feature vectors become more separated in the feature space and this allows better discrimination between the classes [42]. In the present study, the number of dwellings that characterize the stability of predictions over time was significantly lower for moderate compared to low contractions. The better performance of SFS, therefore, reemphasizes the conclusion that the targeted placement might be advantageous when the classification task is more complex. The targeted placement might be particularly relevant for clinical applications, where participants with residual limbs or impaired motor function present overall lower muscle contraction levels and altered muscle structures or recruitment properties [43]. Therefore, clinical rehabilitation might benefit from an electrode configuration that selects the best channels individually for each patient considering their particular condition. However, this hypothesis needs to be tested by conducting assessments in patient populations. Nevertheless, subject-specific targeted placement can be relevant also for general-purpose myoelectric control, for instance, gesture recognition in virtual reality, where low forces might be more natural and lead to better user experience (increased comfort and decreased muscle fatigue). A “smart” matrix electrode can be envisioned in which a small set of pads can be selected and routed to a “low-dimensional” output connector [23]. Such electrodes would allow exploiting the benefits of targeted placement while still using a compact EMG amplifier with only a few input channels. The muscle contraction level did not significantly affect the completion time. Although the prediction was more stable for the moderate level, the participants in this case also needed more time to increase the contraction to reach the target window.

Some recent studies investigated online gesture recognition in combination with wrist movements [26, 35, 36]. In [26], the authors demonstrated that recording EMG from intrinsic muscles improves online gesture classification in a single wrist position. In [35], the authors found that the online classification error produced by changing wrist position could be reduced by placing the electrodes over the intrinsic muscles of the hand. Alternatively, the present study showed that the number of electrodes for classification with changing wrist positions can be substantially reduced without significant loss of performance, but only if the electrodes are strategically placed along the forearm. Containing the channels within the forearm, instead of placing them on the intrinsic hand muscles, simplifies the setup and facilitates the use of myocontrol applications involving hand gesture recognition.

## Conclusion

The present study investigated the selection of electrodes from a high-density matrix required to reach a high accuracy when recognizing hand gestures despite changing wrist positions. The results demonstrated that considering multiple wrist positions indeed increased the complexity of classification, but still high performance could be achieved using only a few electrodes. The online control task showed that targeted, subject-specific placement is important for performance, especially for low levels of muscle contractions. These are relevant insights for the use of myoelectric control, in particular considering the recent trends that regard this approach as an attractive solution for general-purpose human–machine interfacing across applications, domains, and activities.

## Data Availability

Data and materials can be made available upon reasonable request to the authors.

## Abbreviations

ALL: Full set of electrodes
CIRC: Circumferential placement of electrodes
EMG: Electromyography
GUI: Graphical user interface
HD-EMG: High-density electromyography
HO: Hand open
IP: Index pointing
LG: Lateral grasp
MAV: Mean absolute value
LogVar: Logarithm of variance
OP: One-finger pinch
PG: Power grasp
SFS: Electrode placement selected using sequential forward selection
SSC: Slope sign change
TD: Time-domain
TP: Two-finger pinch
TH: Three-finger pinch
WL: Wavelength
ZC: Zero-crossing
